# Effects of Maternal Voluntary Wheel Running During Pregnancy on Adult Hippocampal Neurogenesis, Temporal Order Memory, and Depression-Like Behavior in Adult Female and Male Offspring

**DOI:** 10.3389/fnins.2019.00470

**Published:** 2019-05-21

**Authors:** Suk-Yu Yau, Thomas Ho-Yin Lee, Douglas Affonso Formolo, Wing-Lun Lee, Leo Chun-Kit Li, Parco M. Siu, Chetwyn C. H. Chan

**Affiliations:** ^1^Department of Rehabilitation Sciences, The Hong Kong Polytechnic University, Hong Kong, Hong Kong; ^2^University Research Facility in Behavioral and Systems Neuroscience, The Hong Kong Polytechnic University, Kowloon, Hong Kong; ^3^Divison of Kinesiology, School of Public Health, Li Ka Shing Faculty of Medicine, The University of Hong Kong, Hong Kong, Hong Kong; ^4^Applied Cognitive Neuroscience Laboratory, The Hong Kong Polytechnic University, Kowloon, Hong Kong

**Keywords:** hippocampus, adult neurogenesis, maternal exercise, depression-like behavior, gender, offspring

## Abstract

Research suggests that maternal exercise in pregnancy may have beneficial effects on the brain function of offspring. This study sought to determine if voluntary wheel running during pregnancy improves depression-like behavior, temporal order memory, and hippocampal neurogenesis in both female and male offspring mice. Pregnant mice were allowed to run voluntarily by introducing running wheels into the housing cages throughout the gestational period. Male and female mice offspring at the age of 8- to 9-week-old were then tested on the temporal order task and forced swim test, then euthanized for immunostaining for examining adult hippocampal cell proliferation and neuronal differentiation. Results showed that both male and female pups had reduced depression-like behavior, while only male offspring demonstrated improvement in temporal order memory. Immunostaining revealed that male offspring showed an increase in the number of immature neurons in the ventral hippocampus, whereas female offspring showed enhanced cell proliferation in the dorsal hippocampus. These findings indicate that maternal voluntary wheel running benefits both female and male offspring on reducing depression-like behavior, but with gender effect on promoting hippocampal cell proliferation, neuronal differentiation, and temporal order memory.

## Introduction

Maternal physical exercise during pregnancy lowers the risk of cancer, cardiovascular diseases, and metabolic disorders of the offspring (Blaize et al., 2015). Additionally, maternal exercise can elicit long-lasting and positive effects on the offspring brain during the critical period of fetal brain development (Robinson and Bucci, 2012). In humans, maternal exercise not only improves the growth of fetus and placenta, but also promotes brain development, connectivity, and enhances cognitive functions in offspring in their later life. For example, maternal exercise during pregnancy has been shown to improve intelligence and the language skills of children when they are 5 years old (ClappIII, 1996). Also, maternal physical exercise training including jogging, yoga, weight-lifting, and aerobics during pregnancy promotes language skills in the offspring as assessed when they are 15 months old (Jukic et al., 2013). The results have suggested a long-lasting improvement and transgenerational neuroplasticity induced by maternal exercise in human brains.

The hippocampus is a brain region involved in memory formation and emotion regulation. It is anatomically divided into the dorsal and ventral hippocampi, which are associated with spatial navigation and affective-related functions, respectively (Moser and Moser, 1998). Physical exercise is renowned for improving learning and memory, and reducing depressive behaviors (van Praag et al., 1999a; Sahay and Hen, 2007). These improvements are partly linked to enhanced adult neurogenesis in the hippocampal dentate gyrus (DG) (van Praag et al., 1999b; Kronenberg et al., 2003; Eadie et al., 2005; Erickson et al., 2011). Animal studies have demonstrated that maternal physical exercise enhances cognitive performance and hippocampal neurogenesis in the adult male offspring (Bick-Sander et al., 2006; Lee et al., 2006; Kim et al., 2007; Akhavan et al., 2013; Robinson and Bucci, 2014; Torabi et al., 2017). Moreover, 10 min of forced swimming daily (Lee et al., 2006), 30 min of treadmill running once per day (Kim et al., 2007), or voluntary wheel running (Akhavan et al., 2013; Robinson and Bucci, 2014) thorough the pregnancy period was shown to positively influence the offspring. On the other hand, maternal stress during pregnancy leads to sex-specific detriments in the offspring behavioral performance (Koenig et al., 2005; Schulz et al., 2011; Luine et al., 2017), which is shown to be sex-specific. Similarly, the beneficial effects of maternal physical exercise could also be sex-specific (Titterness et al., 2011). Such sex differences in response to maternal exercise in offspring, however, have not yet been examined.

Taken previous studies together, we hypothesized that maternal voluntary wheel running during pregnancy could improve hippocampal-dependent cognitive performance and hippocampal neurogenesis in a sex-specific manner in the adult offspring. In the present study, we sought to extend the research in this area by Blaize et al. (2015) evaluating the effects of physical exercise during pregnancy on cognitive abilities and depressive behavior in the adult offspring; (Robinson and Bucci, 2012) examining whether behavioral improvements are linked to enhancement in adult hippocampal neurogenesis in the dorsal and ventral DG, and (ClappIII, 1996) determining if there are any sex differences in these effects.

## Materials and Methods

### Animals and Experimental Design

All experimental procedures were approved and followed the guidelines of the Animal Subjects Ethics Sub-Committee from The Hong Kong Polytechnic University. C57BL/6J mice had standard chow and water *ad libitum* in the animal holding room in a 12-h light-dark cycle (lights on at 9 a.m.). Animals were group-housed, as previously described (Yau et al., 2014), in order to avoid the stress induced by social isolation that can affect adult neurogenesis.

**FIGURE 1:**
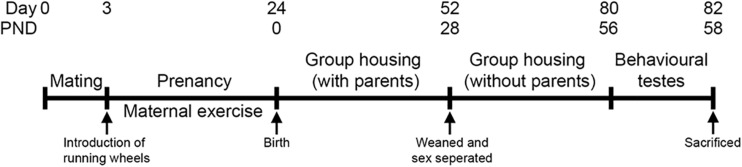
Experimental timeline. Two females were housed with one male in a holding cage. Pregnancy was confirmed by the presence of seminal plugs. After confirming pregnancy, three running wheels were placed in the cages until the pups were born. Pups were weaned and separated on the postnatal day 28. Both male and female offspring were used for the experiments when they aged 8- to 9-week old, and then euthanized at the experimental end point.

Following a 2-day acclimation period, two females were placed with one male. Pregnancy was confirmed by the presence of seminal plugs. After confirmation, three running wheels were introduced in each cage until the pups were born. Pups were weaned, separated from their parents, and placed in different cages according to their sexes on the postnatal day 28 (PND), without running wheels. Male and female offspring at the age of 8- to 9-week old were then subjected to the temporal order task and the forced swim test in two consecutive days, respectively, and were euthanized the day after (Figure 1).

### Behavioral Tests

The adult offspring mice were pre-handled for 7 days to reduce the handling stress as previously performed (Yau et al., 2016). On the next day, they were acclimatized to the behavioral test room for 2 h and then to the empty temporal order testing apparatus for 15 min (Width × Length × Height: 40 cm × 40 cm × 30 cm). Animals were subjected to the temporal order task on the next day, followed by the forced swim test on the day after (only one test was performed per day).

### Temporal Order Task

This test assesses temporal order memory. Mice received three training sessions to explore three copies of a new set of objects (sets 1, 2, 3, respectively) for 5 min, followed by a 5-min intermission interval in transfer cage. During the 5-min test session, mice were allowed to explore a copy of the object set 1 and object set 3. More time spent exploring the first object presented (object set 1 as relatively more novel object) relative to the most recent explored object (object set 3 as recently familiar object) indicates normal temporal order memory. Exploration ratio of the objects was calculated as (exploration time of object 1 or 3)/(exploration time of object 1 + 3). Exploration index was calculated as (exploration time of object 1 – 3)/(exploration time of object 1 + 3) (Yau et al., 2016).

### Forced Swim Test

A mouse was transferred to a cylinder (height: 30 cm; diameter: 15 cm) filled with water at room temperature (24–25°C) and videotaped for 6 min as previously performed (Yau et al., 2014). The time spent immobile during the last 4 min of the testing session was scored by an observer blind to the treatment conditions and presented as an indicator for depression-like behavior. Immobility was defined as any movement beyond what was needed to keep the head above the water (Yau et al., 2014, 2018).

### Tissue Preparation

Mice were deeply anesthetized with isoflurane (1–3% vaporizer, Abbott Laboratories). Upon collection of trunk blood, they were perfused with 0.9% saline and 4% paraformaldehyde (PFA) in 0.01 M phosphate buffered saline (PBS). The brains were post- fixed in 4% PFA overnight at 4°C and were then transferred to 30% sucrose until they sank. The brain sections (1-in-6 series, 30-μm thickness) were obtained coronally using a vibratome (Leica VT1200S). The sections were stored in the cryoprotectant at 4°C until use.

### Immunohistochemical Staining

The free-floating brain sections were retrieved in citrate buffer (pH 6.0) at 95°C for 10 min. After washing in 0.01 M PBS, the sections were incubated overnight with rabbit polyclonal anti-Ki-67 (1:1000; Abcam) antibody at room temperature, and then incubated with biotinylated goat anti-rabbit IgG (1:200; Vector Laboratories, Burlingame, CA, United States) in blocking solution containing 5% normal goat serum for 2 h at room temperature. After three washes in PBS, brain slides were incubated with an avidin-biotin complex (Vector Laboratories) for 2 h as previously described (Gil-Mohapel et al., 2013). The Ki-67 staining was visualized by peroxidase method (ABC system, Vector Laboratories, Burlingame, CA, United States) and diaminobenzidine kits (DAB kits, Sigma-Aldrich, United States) as previously described (Gil-Mohapel et al., 2013). For doublecortin (DCX) staining, sections were incubated with anti-DCX (1:200; Chemicon) antibody at room temperature overnight. After washing three times in PBS, the brain slices were incubated with biotinylated rabbit anti-goat IgG (1:200; Vector Laboratories, Burlingame, CA, United States) in blocking solution containing 5% normal rabbit serum at room temperature for 2 h, followed by three washes in PBS and 2-h incubation with avidin-biotin complex at room temperature. Positive staining was then visualized using the same DAB kit as for the Ki-67 staining.

### Quantification of Ki67^+^ and DCX^+^ Cells

All quantification analyses were performed by a trained researcher using a Nikon series Eclipse H600L microscope. Total number of labeled cells located in the hippocampal DG was counted in a sample blinded manner as previously performed (Gil-Mohapel et al., 2013). Briefly, all DAB-positive cells present within two cell diameters of the subgranular zone (SGZ) were counted. Total number of DCX- or Ki-67-immunopositive cells present in the SGZ of either the dorsal DG (from Bregma −1.34 to −2.54), or the ventral DG (Bregma −2.54 to −3.40) sub-regions were quantified as previously performed (Gil-Mohapel et al., 2013). Total number of positive cells were estimated by multiplying the average number of labeled cells per DG section by the estimated number of 30-μm-thick sections containing either the dorsal or the ventral DG, as previously performed (Gil-Mohapel et al., 2013).

### Statistical Analysis

Independent Student *t*-test was performed to compare between mean values of two experimental groups. To evaluate the effects of maternal voluntary wheel running and sex, a two-way ANOVA followed by Fisher’s *post hoc* test was used. The statistical analyses were performed using the SPSS statistic 25.0 software (SPSS Inc., Chicago, IL). A probability (*P*) value lower than 0.05 was considered statistically significant. Data were shown as means ± SEM.

## Results

### Maternal Voluntary Wheel Running Improved Temporal Order Memory in Male Offspring, but Not Female Offspring

**FIGURE 2:**
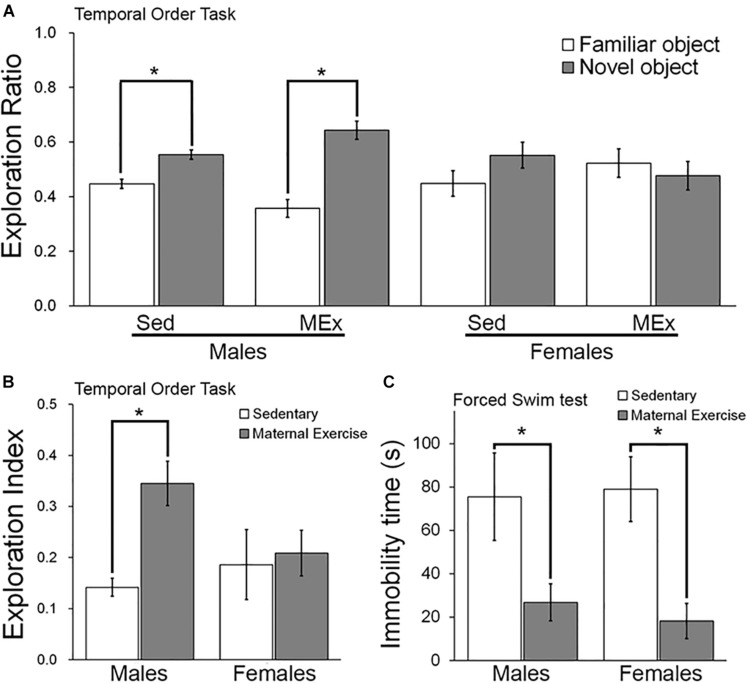
Behavioral performance of offspring in Sedentary (Sed) and Maternal Exercise (MEx) groups. **(A)** Male and female offspring from both Sed and MEx group showed a higher exploration ratio to the novel object than to the familiar object in the temporal order task. **(B)** Male offspring from MEx group displayed a significant enhancement in temporal order memory when compared to Sed group. This effect was absent in female offspring. **(C)** Both male and female MEx pups showed a decrease in depression-like behavior in the forced swim test when compared to Sed pups *p < 0.05; *n* = 5–7 per group.

We first examined the temporal order memory of the offspring. Both male (Figure 2A; *t*_6_ = 9.999 and *t*_10_ = 10.33 respectively) and female offspring (Figure 2A; *t*_8_ = 3.451 and *t*_4_ = 3.809 respectively) showed a significantly greater preference toward the novel object, represented by a longer exploration ratio of the Object 1. In addition, male offspring from the maternal wheel running group had a significant increase in the exploration index, suggesting a better temporal order memory as compared to the sedentary group (Figure 2B; *p* = 0.026). However, the maternal wheel running did not enhance temporal order memory in the female offspring (Figure 2B; *p* = 0.810).

### Maternal Voluntary Wheel Running Reduced Depression-Like Behavior in Both Male and Female Offspring

Two-way ANOVA analysis indicated a significant main effect of maternal exercise on reducing depression-like behaviour in offspring [Figure 2C; *F*(1,20) = 14.45, *p* = 0.001], but no significant main effect of sex [Figure 2C; *F*(1,20) = 0.031, *p* = 0.862]. *Post hoc* analysis demonstrated that maternal exercise significantly reduced depression-like behavior in both male (Figure 2C; *p* < 0.05) and female offspring (Figure 2C; *p* < 0.05). However, there was no interaction between offspring sex and maternal exercise treatment [Figure 2C; *F*(1,20) = 0.179, *p* = 0.677].

### Maternal Voluntary Wheel Running Increased the Number of Immature Neurons in Ventral DG, but Not Dorsal DG of Male Offspring

**FIGURE 3:**
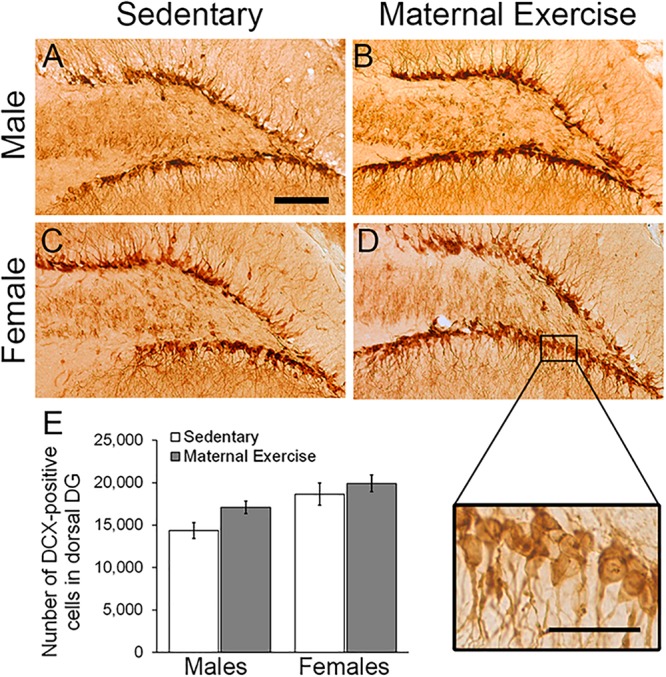
Effect of maternal exercise on number of immature neurons in the dorsal DG of offspring. Representative images of DCX positive cells in the dorsal DG **(A,C)** sedentary control and **(B,D)** maternal exercise group at 20 × (scale bar = 100 μm) and 40 × (scale bar = 50 μm) magnification. **(E)** No significant effect of maternal exercise on the number of immature neurons in the offspring was observed when compared to sedentary control in both sexes. *n* = 5–7 per group.

**FIGURE 4:**
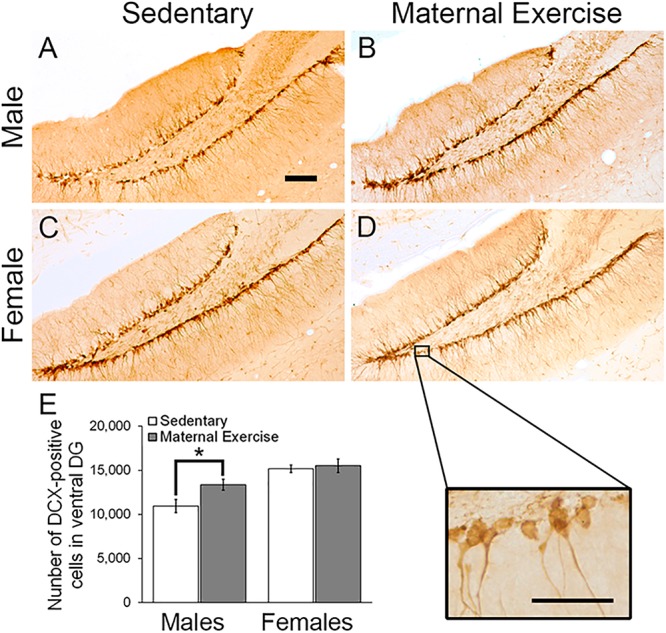
Effect of maternal exercise on number of immature neurons in the ventral DG of offspring. Representative images of DCX positive cells in the ventral DG **(A,C)** sedentary control and **(B,D)** maternal exercise group at 20 × (scale bar = 100 μm) and 40 × (scale bar = 50 μm) magnification. **(E)** Maternal exercise significantly increased the number of immature neurons in male offspring when compared to male sedentary control, while this effect was not observed in females. *P < 0.05; *n* = 5–7 per group.

Two-way ANOVA analysis revealed that maternal exercise did not increase the number of immature neurons in the dorsal DG in both male and female offspring [Figure 3; main effect of exercise: *F*(3,23) = 3.982, *p* = 0.060, main effect of sex: *F*(3,23) = 12.594, *p* = 0.002]. However, there was a significant main effect of sex on increasing the number of immature neurons in the ventral DG [Figures 4A–E; *F*(3,23) = 18.017, *p* = 0.0004]. *Post hoc* test revealed that maternal exercise significantly increased the number of DCX positive cells in the ventral DG of the male offspring when compared to the sedentary group (Figure 4E; *p* < 0.05), but not in the females (*p* = 0.810).

### Maternal Voluntary Wheel Running Increased Hippocampal Cell Proliferation in Dorsal, but Not Ventral DG of Female Offspring

**FIGURE 5:**
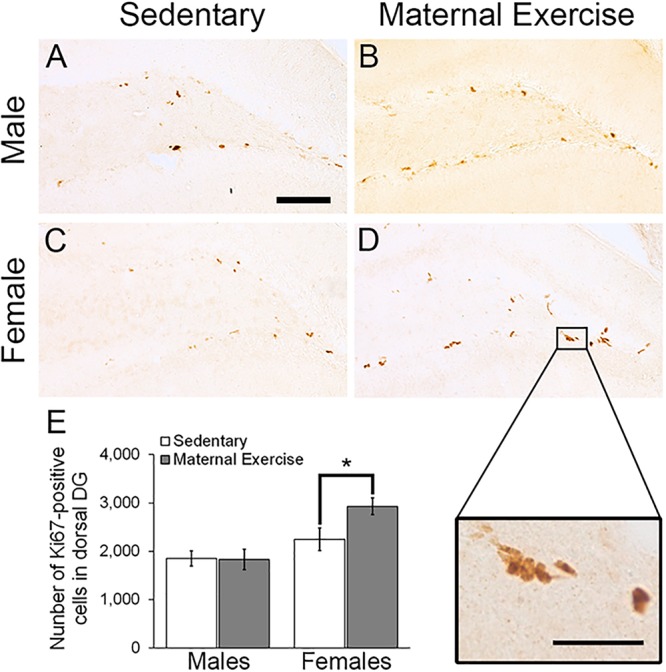
Effect of maternal exercise on cell proliferation in the dorsal DG of offspring. Representative images of Ki-67 positive cells in the dorsal DG from **(A,C)** sedentary control and **(B,D)** maternal exercise group at 20 × (scale bar = 100 μm) and 40 × (scale bar = 50 μm) magnification. **(E)** Maternal exercise significantly increases cell proliferation in dorsal DG of female offspring when compared to female sedentary control, while this effect was not observed in male offspring. *P < 0.05; *n* = 5–7 per group.

**FIGURE 6:**
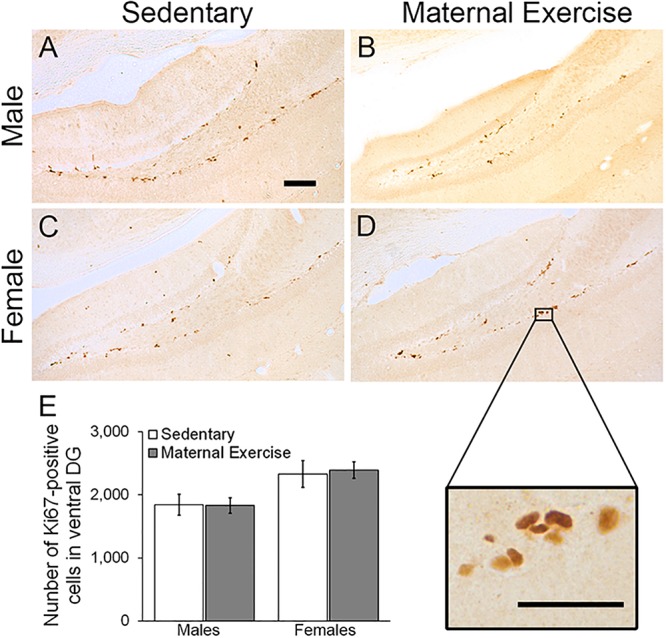
Effect of maternal exercise on cell proliferation in the ventral DG of offspring. Representative images of Ki-67 positive cells of **(A,C)** sedentary control and **(B,D)** maternal exercise group at 20 × (scale bar = 100 μm) and 40 × (scale bar = 50 μm) magnification. **(E)** Number of proliferating cells in ventral DG of offspring from sedentary and maternal exercise group were comparable in both sexes. *n* = 5–7 per group.

Maternal exercise increased the number of the dorsal DG proliferating cells in sex-specific manner, as represented by a main effect of sex in the two-way ANOVA [Figures 5A–E; main effect of exercise: *F*(3,22) = 2.036, *p* = 0.170, main effect of sex: *F*(3,22) = 10.470, *p* = 0.004]. *Post hoc* analysis showed that maternal exercise increased the dorsal DG cell proliferation levels in the female offspring (Figure 5E; *p* < 0.05), but not in the males (Figure 5E; *p* = 0.950). Maternal exercise did not show significant effect on the ventral DG [Figure 6; *F*(3,23) = 2.657, *p* = 0.076], either for male (*p* = 0.961) or female offspring (*p* = 0.808) when compared to control.

## Discussion

Our results demonstrated that maternal exercise during pregnancy, in terms of voluntary wheel running, significantly reduced depression-like behavior in both adult male and female offspring. Interestingly, temporal order memory improved only in the male offspring in concurrent with a significant increase in the number of immature neurons in the ventral DG. In contrast, maternal voluntary wheel running increased the number of proliferating cells in the dorsal DG of the female offspring, without affecting the temporal order memory performance.

Previous rodent studies have documented the benefits of voluntary wheel running on cognitive function and hippocampal neurogenesis (van Praag et al., 1999b; Kronenberg et al., 2003; Eadie et al., 2005; Erickson et al., 2011). An earlier study revealed that the beneficial effect of physical activity could be passed from the mother to their progeny (Bick-Sander et al., 2006). Our investigation extends these findings by showing that maternal physical activity improved hippocampal cell proliferation and neuronal differentiation as well as cognitive functions in the adult offspring. The current data is also consistent with previous reports showing the positive impacts of exercise on depression-like behavior, learning and memory that persisted into adulthood (Bick-Sander et al., 2006; Lee et al., 2006; Kim et al., 2007; Dayi et al., 2012; Akhavan et al., 2013; Torabi et al., 2017). However, Dayi et al.’s (2012) study showed improved spatial learning memory, as indicated by the Morris water maze test, in both male and female adult offspring. Contradicting that, in our study only the male offspring showed improvement in the hippocampal-dependent memory, as reflected by a better performance in the temporal order task. Such contradictions could be explained by differences in the tasks used in each study, as well as in the specific maternal exercise protocols used.

In spite of the multiplicity of exercise training paradigms, including forced treadmill running (Bick-Sander et al., 2006; Kim et al., 2007), forced swimming (Lee et al., 2006), and voluntary wheel running (Akhavan et al., 2013; Robinson and Bucci, 2014), the beneficial effects of maternal exercise on offspring have been consistently reported. Here, the adoption of the voluntary wheel running paradigm was motivated by a lower stressful burden that it imposes to the mothers, whereas a more stressful form of exercise, namely the forced treadmill running, was used in Dayi et al.’s (2012) study. The effects of exercise on brain development could depend on the intensity, duration, and the frequency of the exercise (Speck et al., 2014). However, in order to avoid stress caused by social isolation to pregnant moms, we adopted a paired housing condition with a shared running wheel. In doing so, we could not accurately measure the individual running activity of maternal mice during pregnancy. Since physical voluntary wheel running is performed by pregnant mom, male and female offspring from the same litter were under the influence of the same amount of running activity from the pregnant dam. It warrants further investigation on the correlations between the amount of physical activity in pregnant dams and the levels of hippocampal adult neurogenesis and behavioral phenotype in the adult male and female offspring.

In addition to the improvements in cognitive performance, we also investigated the effect of maternal exercise on the proliferation of hippocampal progenitor cells and the number of immature neurons in the adult offspring. Male offspring with maternal exercise showed an increased number of immature neurons as indicated by DCX staining in the ventral DG, whereas female offspring showed an increased number of proliferating cells as indicated by Ki-67 staining in the dorsal DG. A previous study has indicated that the ventral hippocampus is specifically responsible for modulating emotional and affective processes, whereas the dorsal hippocampus is involved in spatial learning and memory formation (Fanselow and Dong, 2010). Particularly, changes in hippocampal neurogenesis in the ventral DG could be associated with depression phenotype, as evidenced by a study showing that selective enhancement of neurogenesis in the ventral DG could reduce depression-like behavior in rodents (Banasr et al., 2006). In the present study, the results suggest that the decrease in depressive-like behavior observed in the male offspring might be partly linked to an increase in neuronal differentiation in the ventral DG. In contrast, a decrease in depressive-like behavior in females could be independent of changes in the ventral DG neurogenesis, but linked to other mechanisms. In addition to enhancement in adult neurogenesis, it is possible that maternal exercise may induce other forms of neuroplasticity leading to reduced depression-like behavior in the offspring. Animal studies have demonstrated that maternal exercise increases hippocampal levels of the brain-derived neutrophic factor (BDNF) (Liu and Nusslock, 2018), a factor important for promoting structural and functional plasticity. We have previously shown that both dendritic remodeling in the hippocampal CA3 subregion and enhancement in adult neurogenesis in the dentate region are required for the antidepressant effects of voluntary running in a rat model of depression (Yau et al., 2011). Furthermore, running can significantly enhance long-term potentiation in the hippocampal DG in mice (van Praag et al., 1999a). Enhanced dendritic complexity and synaptic plasticity may therefore also contribute to the improvement of depression-like behavior in both male and female offspring. Also, a previous study using C57BL/6J mouse strain revealed that voluntary wheel running elicits differential effects on synaptic plasticity of male and female hippocampi (Titterness et al., 2011), warranting further investigation on whether maternal voluntary wheel running can elicit transgenerational benefits in terms of dendritic complexity and synaptic plasticity in both female and male offspring.

Studies have also shown that maternal stress during pregnancy differently affect male and female offspring in terms of hippocampal-dependent behavioral tasks, suggesting that males may be more sensitive to gestational stress than females (Bowman et al., 2004; Koenig et al., 2005; Weinstock, 2005). Similarly, our results showed that male offspring had a significant improvement in a hippocampal dependent memory, which was absent in females. It may suggest that male offspring could be more sensitive to maternal exercise than females in terms of hippocampal dependent learning and memory tasks. However, as previously stated, further studies examining the effects of different intensities of maternal running on hippocampal dependent learning and memory tasks, as well as hippocampal adult neurogenesis in female and male offspring will be of great interest. Such study will further confirm whether there is a dosage effect in response to the maternal exercise-induced increase in hippocampal neurogenesis in the offspring.

In addition, a gender-specific effect was observed with an increased number of immature neurons in the ventral DG of male offspring, and an increased number of proliferating cells in the dorsal DG of female offspring. Indeed, it has been previously shown that voluntary exercise elicits differential effects on synaptic plasticity of the male and female hippocampus (Titterness et al., 2011). It is known that sex hormones including progesterone, testosterone, and estradiol have differential effects on modulating hippocampal adult neurogenesis and dendritic complexity in male and female rodent brains (Trivino-Paredes et al., 2016). An earlier study reported that ovariectomy abrogated the exercise-induced up-regulation of BDNF mRNA level in female rats (Berchtold et al., 2001), suggesting a potential role of endogenous gonadal hormones on influencing hippocampal plasticity. Because an increase in testosterone promotes cell survival in males, but not female animals (Farinetti et al., 2015), it is possible that differences in sex hormones between males and females contribute to the sex-specific response to maternal exercise. Furthermore, we obtained the offspring from seven different litters in total. Given that there was usually a small number of pups, which were born from the first litter from the naïve dam, we only included two to four offspring from each litter for the experiment. The litter effect as a confounding factor should be considered since the litter effect could have influenced the reported results. Future studies should include two animals from each litter in a given experimental group to ensure the potential litter effects will not influence the overall results.

Despite the limitations, we have demonstrated novel findings regarding the benefits of maternal exercise on offspring and its effect on modulating hippocampal cell proliferation and neuronal differentiation of new-born cells in a sex-specific manner. Specifically, we identified differential effects of maternal voluntary wheel running on the adult offspring, with a sex-specific effect on enhancing learning and memory performance in male, but not female offspring. This behavioral benefit was partly linked to increased neuronal differentiation of new-born neurons. The current findings provide the first evidence that male and female offspring respond differently to maternal exercise. The mechanisms underlying the sex-specific effects of maternal exercise on the offspring require future studies.

## Data Availability

The datasets for this manuscript are not publicly available because there is not a link or online drive to share the data yet at my institute. Requests to access the datasets should be directed to sonata.yau@polyu.edu.hk.

## Ethics Statement

This study was carried out in accordance with the recommendations of guideline by the central animal facilities at Hong Kong Polytechnic University. The protocol was approved by the animal subjects ethics committee.

## Author Contributions

S-YY, LC-KL, and W-LL performed the experiments. S-YY, LC-KL, TH-YL, and DAF analyzed the data. S-YY, LC-KL, TH-YL, DAF, PS, and CC prepared the manuscript.

## Conflict of Interest Statement

The authors declare that the research was conducted in the absence of any commercial or financial relationships that could be construed as a potential conflict of interest.
